# Imported mixed malaria caused by *Plasmodium falciparum* and *Plasmodium ovalecurtisi*: An Italian case report

**DOI:** 10.1007/s00436-026-08778-z

**Published:** 2026-10-06

**Authors:** Cataldo Maria Mannavola, Gianluca Quaranta, Vanessa Di Pietro, Valentina Siciliano, Giovanni Fancello, Cristina De Vivo, Giuseppe Maiuro, Antonella Cingolani, Maurizio Sanguinetti, Carlo Torti, Luca Masucci

**Affiliations:** 1 Department of Basic Biotechnological Sciences, Intensive and Perioperative Clinics, Università Cattolica del Sacro Cuore, Rome, Italy; 2 Department of Laboratory and Hematological Sciences, Fondazione Policlinico Universitario A. Gemelli IRCCS, Rome, Italy; 3 Department of Medical and Surgical Sciences, Fondazione Policlinico Universitario A Gemelli IRCCS, Rome, Italy; 4 Department of Safety and Bioethics, Infectious Diseases Section, Università Cattolica del Sacro Cuore, Rome, Italy

**Keywords:** Mixed *Plasmodium* infection, *Plasmodium ovalecurtisi*, Molecular diagnosis

## Abstract

Malaria remains a major global health threat, particularly in endemic regions of sub-Saharan Africa. In 2024 alone, an estimated 282 million cases were reported worldwide, with approximately 610,000 associated deaths. Beyond the high disease burden, mixed-species *Plasmodium* infections represent an additional clinical challenge, as they are frequently underdiagnosed and may significantly complicate both diagnostic accuracy and therapeutic management even in non-endemic countries. We report the case of a 67-year-old man from Guinea who presented to the Emergency Department with high-grade fever and asthenia shortly after returning to Italy; initial microbiological investigations were negative, despite markedly elevated inflammatory markers were diagnosed. The patient reported a previous untreated malaria episode approximately six months earlier, raising suspicion of a novel *Plasmodium* infection. A comprehensive malaria diagnostic workflow was promptly initiated, integrating rapid immunoenzymatic testing (IE), microscopy, and molecular assays. The IE test suggested *Plasmodium falciparum* or a mixed infection as result, while loop-mediated isothermal amplification (LAMP-PCR) confirmed the presence of *Plasmodium spp* DNA. Microscopic examination of stained blood smears revealed typical *P. falciparum* ring forms and suspected *P. ovale* trophozoites; so, species-specific real-time PCR confirmed a mixed infection with *P. falciparum* and *P. ovale*, supporting prompt antimalarial treatment. Final molecular confirmation was achieved by Sanger sequencing, which identified the *Plasmodium ovalecurtisi* as co-aetiological agent of disease. The rapid and integrated diagnostic approach enabled timely initiation of appropriate antimalarial therapy with artemether–lumefantrine and primaquine, leading to complete clinical recovery and gene sequencing allowed a correct species confirmation and epidemiological characterization. This case highlights the importance of combining microscopic analysis with advanced molecular diagnosis for an accurate identification of mixed *Plasmodium* infections even in country and contributes novel epidemiological data through molecular characterization of *P. ovalecurtisi* in an imported case.

**Supplementary Information:**

The online version contains supplementary material available at https://doi.org/10.1007/s00436-026-08778-z.

## Introduction

Despite substantial progress in prevention and treatment strategies, malaria continues to represent a major public health challenge, particularly in African settings (WHO 2025). This disease remains responsible for a considerable burden of morbidity and mortality, with hundreds of millions of cases reported annually worldwide (Li et al. 2025).

Mixed-species *Plasmodium* infections are sometimes recognized in imported malaria cases and pose significant diagnostic and therapeutic challenges, especially in non-endemic settings (Marteau et al. 2021): these infections are frequently underdiagnosed due to low parasitemia levels and overlapping morphological features on light microscopy, potentially leading to delayed or incomplete treatment (Marteau et al. 2021, Picot et al. 2020).

Although molecular diagnostic techniques have improved the detection of mixed infections, complex mixed-species *Plasmodium* infections are often challenging to diagnose and manage in non-endemic areas, where diagnostic approaches may vary according to local expertise and laboratory availability (Daily et al. 2022). Here, we describe a case of imported malaria caused by a mixed infection with *Plasmodium falciparum* and *Plasmodium ovalecurtisi*, diagnosed through an integrated diagnostic workflow combining microscopy, molecular assays, and gene sequencing.

## Case history

A 67 year-old man, resident in Guinea and temporarily staying in Rome, presented to Emergency Department (ED) of Fondazione Policlinico Universitario A. Gemelli IRCCS in Rome on August 2025 with shiver fever and asthenia. His recent clinical history dated back to July, when he first developed fever accompanied by chills and asthenia, without additional symptoms. Upon arrival to the ED, his body temperature was 39 °C high; laboratory tests revealed leukopenia (blood cells 3,700/mm³), with neutrophils accounting for 3,080/mm³; serum glucose was 119 mg/dL; hemoglobin was 14.4 g/dL at admission and decreased to 12.1 g/dL approximately 24 h later. while aspartate aminotransferase was markedly elevated at 71 U/L, procalcitonin and C-reactive protein levels were significantly increased at 7.28 ng/mL and 162.5 mg/L, respectively, indicating an ongoing systemic inflammatory response.

Due to the markedly elevated inflammatory markers, blood cultures were also obtained to investigate a possible bloodstream infection which later returned negative.

Given the patient’s recent travel history and clinical presentation, a rapid multiplex molecular panel for neurotropic arboviruses, including Dengue virus, Zika virus, Chikungunya virus, West Nile virus, and yellow fever virus (FilmArray^®^ Meningitis/Encephalitis Panel, BioFire Diagnostics, Salt Lake City, UT, USA), was performed and returned negative.

The patient reported an episode suggestive of malaria approximately six months earlier in Guinea, for which he reported having received no antimalarial treatment. No medical or laboratory documentation of this previous episode was available; therefore, diagnostic confirmation of malaria at that time could not be established. Considering this history, a comprehensive malaria diagnostic workup was subsequently initiated at our centre, as is routine for any suspected malaria parasite infection.

First, a blood sample was collected; from this sample, at least three blood smears were immediately prepared and subsequently stained with May-Grunwald-Giemsa (Marteau et al. 2021). Another aliquot of the blood was used for an immunoenzymatic rapid assay (Malaria CareUS Rapydtest^®^, Wokingham, UK; IE) to obtain a rapid indication of malarial infection. In parallel, a loop-mediated isothermal amplification polymerase chain reaction (LAMP-PCR; Illumipro-10, Meridian Bioscience Inc., Cincinnati, USA) was performed in about 1 h, providing a rapid yet more sensitive assessment of *Plasmodium* spp. infection.

The IE test indicated a result consistent with “*Plasmodium falciparum* or mixed infections,” as indicated by manufacturer’s instructions, while the LAMP-PCR assay detected a positive *Plasmodium* spp. infection. Thus, our rapid tests confirmed the presence of at least one *Plasmodium* species in about 1 h.

**Fig. 1:**
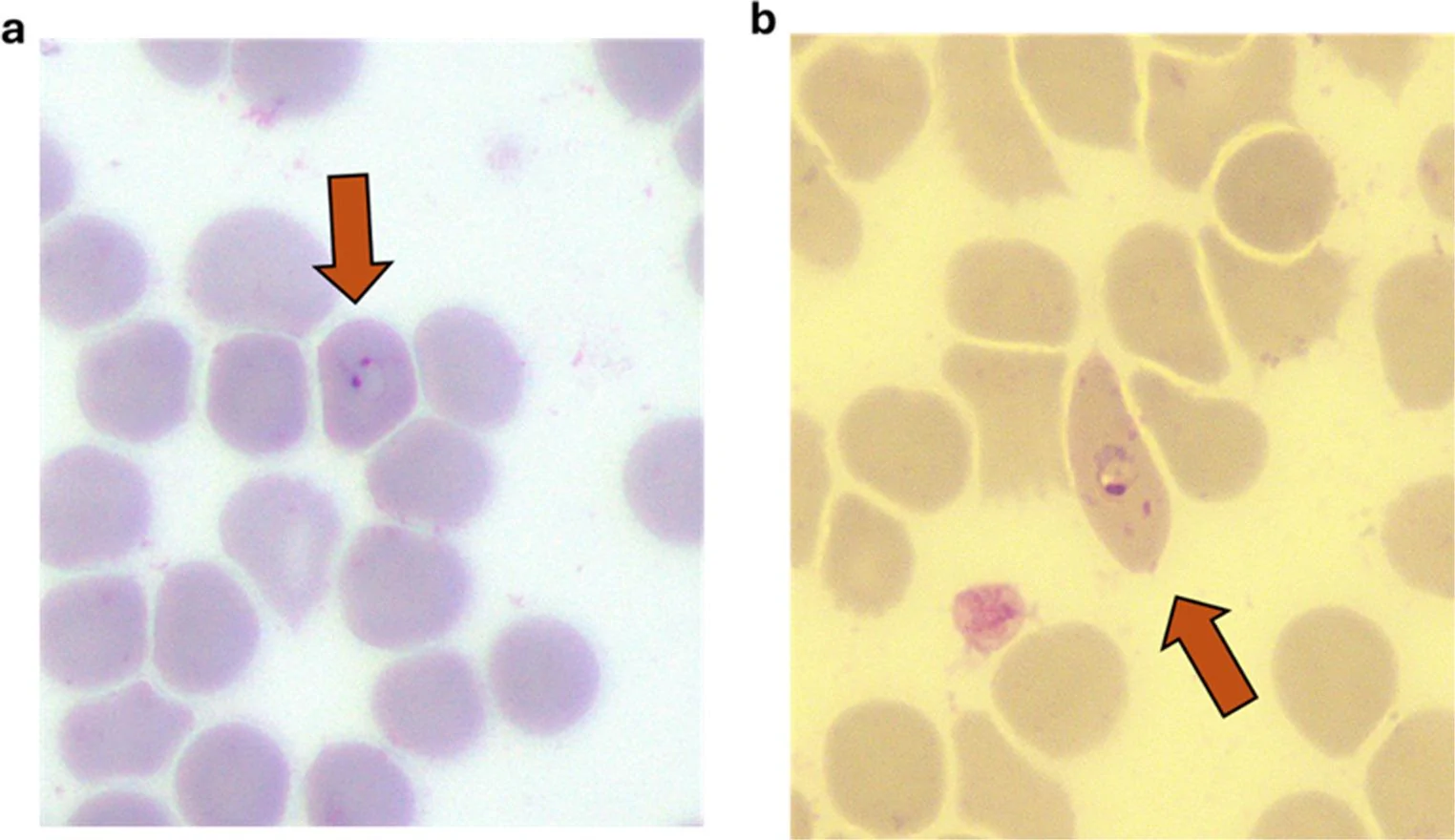
**(a**) Microscopic examination of *P. falciparum* trophozoites (May–Grünwald–Giemsa stain, 100x objective magnification) showing the characteristic ring forms; (**b**) Microscopic examination of *P. ovale* trophozoites (May–Grünwald–Giemsa stain, 100x magnification), with the typical oval-shaped infected erythrocytes from which the species derives its name

Microscopic examination additionally revealed several *P. falciparum* trophozoites (Fig. 1, a), as well as forms suspected to belong to *P. ovale* (Fig. 1, b), with a total parasitaemia of < 1% under light microscopy, using an Olympus BX60 microscope (Olympus, Japan) with a 100 X objective. Although *Schüffner’s* stippling was not clearly appreciable, the observed oval-shaped erythrocytes and trophozoite morphology were highly suggestive of *P. ovale* infection. Specifically, *P. falciparum* parasitaemia was estimated at 0.4%, while *P. ovale* parasitaemia was approximately 0.15%. Due to these microscopic findings and the positive but ambiguous IE test result, a real-time PCR (RT-PCR; VIASURE Real Time PCR Detection Kits, Certest Biotec, San Mateo de Gállego, Zaragoza, Spain) was performed for its greater sensitivity; DNA was manually extracted from 200 µL of whole blood using the High Pure PCR Template Preparation Kit (Roche, Basel, Switzerland) and eluted in a final volume of 200 µL (1:1 ratio). Subsequently, 5 µL of the extracted DNA was used as template for the RT-PCR assay which finally revealed a positivity for *P. falciparum* (Ct = 30.86) and *P. ovale* (Ct = 37.33), confirming the microscopic observation in May-Grunwald-Giemsa stain.

Moreover, for final molecular confirmation, we performed a nested PCR using two specific primer pairs followed by Sanger gene sequencing (Snounou et al. 1993), in order to type Plamodium ovale. Ultimately, gene sequencing confirmed the presence of *P. ovalecurtisi*.

Following the first line diagnostic results, obtained within approximately 2 h and strongly suggestive of *Plasmodium falciparum* infection, antimalarial therapy with artemether–lumefantrine was promptly initiated. Subsequent molecular investigations, completed within two days, confirmed the mixed infection with *P. falciparum* and *P. ovalecurtisi*, supporting the addition of primaquine to the therapeutic regimen (Glucose-6-phosphate dehydrogenase deficiency was excluded before primaquine administration). The patient was discharged in good general condition, afebrile, with progressive improvement in inflammatory markers.

## Discussion and results

Malaria is an infectious disease caused by protozoa parasites belonging to the genus of *Plasmodium* (Daily et al. 2022); in particular, they are transmitted from female A*nopheles* mosquitoes which acquire the parasites during a blood meal from an infected host and subsequently transmit them to new hosts through additional mosquito bites (Daily et al. 2022). Five *Plasmodium* species are responsible for human malaria: *P. falciparum*, the most common, *P. vivax*, *P. ovale*, *P. malariae*, and *P. knowlesi* (Li et al. 2025). Rare cases of non-vector transmission have also been documented, including vertical transmission during pregnancy or at birth, transmission through blood transfusion, and infections resulting from needle sharing (Daily et al. 2022).

Malaria remains a critical global health threat: in 2024, an estimated 282 million cases were reported worldwide (WHO 2025); in the same year, approximately 610,000 malaria-related deaths were recorded, representing an increase of 12,000 compared with 2023, particularly driven by rises in Madagascar, Yemen, and Ethiopia (World Health Organization 2025).

In this manuscript, we describe a 67-year-old Guinean man presenting with febrile illness and a history of untreated malaria approximately six months earlier. First-line investigations suggested a *P. falciparum* or mixed infection, while microscopic examination revealed typical *P. falciparum* ring forms together with highly suspicious forms compatible with *P. ovale* (Fig. 1a and b), prompting further molecular characterization (Shin et al. 2020).

Successively, to achieve species-level confirmation a RT-PCR was performed, which showed a mixed infection with both *P. falciparum* and *P. ovale*. Antimalarial treatment based on artemether-lumefantrine was initiated promptly after first-line diagnostic confirmation in ≈ 2 h, while molecular characterization was completed subsequently for species-level confirmation and epidemiological purposes in 2 days total.

**Fig. 2:**
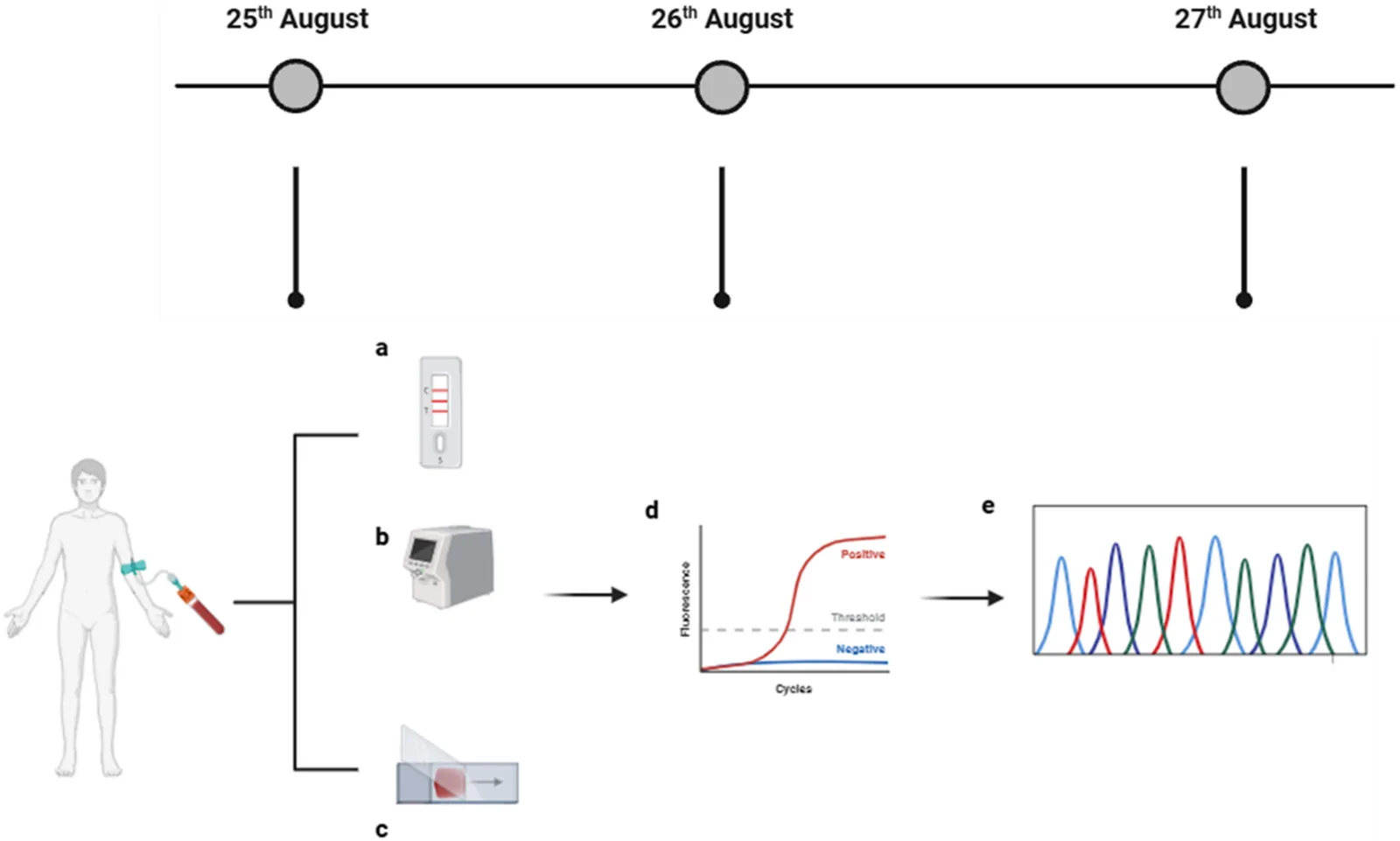
Schematic diagnostic workflow for malaria parasite infection. A patient with suspected malaria was admitted to the ED, and a series of diagnostic investigations were performed on a blood sample: **a**) an IE rapid test, which indicated *P. falciparum* or mixed infections (three coloured bands) within approximately 10 minutes; **b**) a LAMP-PCR assay, which detected the presence of *Plasmodium* spp. DNA within approximately 1 h. **c**) In parallel with tests **a **and **b**, a May–Grünwald–Giemsa-stained thin blood smear was prepared, representing a pivotal component of the diagnostic workflow. **d**) Following the positive results from **a **and **b**, and the microscopic detection of P. falciparum trophozoites together with suspected P. ovale forms, a second-line molecular test (RT-PCR) was performed, confirming *P. falciparum* (Ct = 30.86) and *P. ovale* (Ct = 37.33) detection. **e**) Sanger gene sequencing wich yield to *P. ovalecurtisi* as result

The entire diagnostic workflow is schematised in Fig. 2.

Dealing with local epidemiology, the World Health Organization (WHO) did not include a specific country profile for Guinea in its latest report, owing to limited and inconsistent data availability related to logistical and technical constraints. However, in 2023 the WHO estimated that Guinea reported approximately 4.4 million malaria cases (WHO 2025); due to substantial regional disparities within the country, detailed epidemiological analyses describing the distribution and burden of malaria species are not available, so national data remain incomplete.

Nevertheless, a local Guinean survey from 2012 reported that, as in many other African regions, *P. falciparum* remains the predominant species responsible for malaria parasite infections (98%), whereas *P. ovale* accounts for a much smaller proportion (0.5%) (Institut national de la Statistique 2021). Moreover, *P. ovale* infections were more frequently detected as part of mixed infections rather than as mono-infections (Mahittikorn et al. 2021), consistent with the case-presentation described in this manuscript. Therefore, based on the available epidemiological evidence, our case aligns with the expected distribution of *Plasmodium* species within the Guinean context.

The main feature of the *P. ovale* life cycle is its hypnozoite stage in the human liver, where the parasite may remain dormant for weeks to years before reactivating and developing into hepatic merozoites, then entering into the bloodstream and initiating a blood-stage infection (Daily et al. 2022). It is likely that our patient had been infected several months earlier during his previous malaria episode; however, an inconsistent and non-specific diagnostic workup may have failed to detect the presence of *P. ovale*, which could have persisted in the liver in a dormant state, possibly facilitated by inappropriate or incomplete therapy. After approximately six months, in presence of permissive exogenous factors and host conditions, *P. ovale* may have reactivated and be able to start a new blood-stage infection. As demonstrated by Kotepui et al., *P. ovale* infections carry a lower risk of progressing to severe disease compared with *P. falciparum*, which remains the most virulent *Plasmodium* species in malaria pathogenesis (Kotepui et al. 2020). Nevertheless, *P. ovale* may cause severe malaria, although typically with less pronounced clinical alterations than *P. falciparum*, particularly manifesting as jaundice, severe anemia, and pulmonary impairment (Kotepui et al. 2020). In all cases, the recommended therapy for mixed infections involving *P. ovale*, as suggested for severe *P. falciparum* infections is artesunate and lumefantrine (in combination with artemether) and primaquine to target the erythrocytic stage of infection (Aniweh et al. 2023). In the present case, molecular characterization did not alter the initial antimalarial management, which was promptly initiated based on first line diagnostic findings, but provided additional species-level and epidemiological information regarding the *P. ovale* isolate.

Indeed, as reported by Snounou et al. in a recent opinion article, *P. ovale* strains are divided up into *wallikeri* and *curtisi*, which represent two genetically distinct and non-recombining species (Snounou et al. 2024); accordingly, the previously used trinomial nomenclature, which has sometimes led to confusion, has been replaced by a binomial nomenclature recognizing *P. ovalecurtisi* and *P. ovalewallikeri* as separate species. For this reason, we adopted this nomenclature throughout the present manuscript.

Dealing with malaria diagnosis, microscopic observation remains the gold standard, although it requires an experienced physician and a limit of detection between 50 and 500 parasites/µL (Kotepui et al. 2020); indeed, in some cases, *P. ovale* forms may be misidentified as *P. vivax* (Kotepui et al. 2020). In this context, rapid diagnostic tests such as IE and molecular approaches have been developed to address the sensitivity and time limitations of microscopic detection, allowing the recognition of low amounts of circulating *Plasmodium* antigens and DNA also in not specialistic settings (Bigaillon et al. 2005, Marx et al. 2005). The IE test used in this case report is able to detect *P. falciparum* histidine-rich protein 2 (HRP2) antigen and pan-parasite lactate dehydrogenase (pLDH), which is shared by *P. falciparum*, *P. ovale*, *P. vivax* and *P. malariae*. Overall, IE tests are not sufficiently effective for the diagnosis of *P. ovale*, as they exhibit low overall sensitivity; however, pLDH-based tests have been reported to be more accurate in detecting *P. ovalewallikeri* than *P. ovalecurtisi* (Joste et al. 2021): this difference may be explained by polymorphisms in *P. ovale* LDH, which could affect antibody affinity in IE assays (Joste et al. 2021). In light of these data, the IE test used in this diagnostic workflow was able to correctly identify a suspected mixed infection, even though it is well known that the performance of rapid diagnostic tests may be influenced by multiple factors, including parasite density, host-related factors and the timing of laboratory diagnosis (Ojeniyi et al. 2025, Martiáñez-Vendrell et al. 2022).

Moreover, molecular tests such as PCR, enable detection of low quantities of various *Plasmodium* spp. DNA, even in doubtful cases or where microscopy suggests a non-*P. falciparum* species infection (Kotepui et al. 2020, Calderaro et al. 2007).

Recent evidence shows that molecular approaches provide very high sensitivity and specificity compared to microscopy in malaria diagnosis (Picot et al. 2020); for this reason, molecular assays may provide useful support in selected cases of imported malaria, particularly for species confirmation, mixed infections and epidemiological characterization, as performed in this manuscript (Picot et al. 2020).

Although RT-PCR confirmed the mixed infection (*P. ovale*, Ct = 37.33; *P. falciparum*, Ct = 30.86), these Ct values cannot be directly correlated with microscopically determined parasitemia (Perandin et al. 2004). The apparent discrepancy may reflect the operator-dependent nature of microscopic quantification and sampling variation between blood aliquots used for microscopy and molecular analysis. Moreover, the assay is primarily optimized for qualitative detection, while quantitative interpretation requires the manufacturer-provided Malaria Quantitative Standard and remains influenced by interspecies variability in 18 S rRNA gene copy number (Ulloa et al. 2025). Thus, molecular techniques are valuable for confirming low-density and mixed infections, whereas microscopy remains the gold standard for parasitemia determination (Centers for Disease Control and Prevention 2024).

Additionally, we decided also to enhance our diagnostic algorithm in this case by sequencing the *P. ovale* strain, because diagnostic workflows for imported malaria may vary according to local expertise and laboratory availability, although evidence remains limited and sometimes conflicting, we decided to perform Sanger gene sequencing to characterize the *P. ovale* strain (Liew et al. 2016). Unfortunately, no epidemiological reports specifically addressing even *P. ovale* subspecies in Guinea are available, likely due to the lack of population-based studies and surveillance programs in some area of the region.

So, a nested-PCR was performed, using two specific primer pairs amplifying the small subunit ribosomal RNA of *Plasmodium*, as described (Snounou et al. 1993). The final PCR products indicated even in this case a co-infection of *P. falciparum* and *P. ovale* (Supplementary figure S1, line 1 and 10). The positive *P. ovale* product was subsequently prepared for Sanger sequencing (Cosio et al. 2025), which confirmed *P. ovalecurtisi* as result; gene sequence has then been deposited in GenBank under the accession number PX920863.

Although epidemiological data from Guinea are lacking, evaluations performed on imported cases from Guinea suggest a slight predominance of *P. ovalecurtisi* in the region (Zhang et al. 2021): in this context, the *P. ovalecurtisi* strain reported in this case aligns with previously reported observations in literature.

Although mixed *Plasmodium* infections have been identified in imported malaria cases in Italy using molecular methods, to our knowledge there are no previous Italian case reports of a *P. falciparum* and *P. ovalecurtisi* co-infection confirmed by microscopic identification, molecularly characterized and with sequence deposition in public databases.

In conclusion, we report a well-documented case of mixed infection with *Plasmodium falciparum* and *Plasmodium ovalecurtisi* in an imported malaria case, promptly diagnosed and successfully treated with complete clinical recovery. This case highlights the value of integrating microscopy with molecular methods for the confirmation and characterization of mixed-species *Plasmodium* infections, particularly in non-endemic settings.

In our patient molecular assays and Sanger sequencing did not modify the initial antimalarial management, which was promptly initiated based on first line diagnostic results, but provided accurate species level characterization of the *P. ovale* isolate and additional epidemiological information. The deposition of the sequence in a public database may further contribute to the molecular epidemiology of imported malaria cases from Guinea and to the growing knowledge regarding the *P. ovale complex*.

## Supplementary Information

Below is the link to the electronic supplementary material.

Supplementary Material 1

## Data Availability

No datasets were generated or analysed during the current study.

## Abbreviations

ED: Emergency Department
HRP2: Histidine-Rich Protein 2
IE: Immunoenzymatic Assay
LAMP-PCR: Loop-Mediated Isothermal Amplification Polymerase Chain Reaction
PCR: Polymerase Chain Reaction
pLDH: Plasmodium Lactate Dehydrogenase
RT-PCR: Real-Time Polymerase Chain Reaction
WHO: World Health Organization
