# Supplementary material for: Imported mixed malaria caused by Plasmodium falciparum and Plasmodium ovalecurtisi: An Italian case report
Source: Parasitol Res. 2026 Oct 6;125(1):131. doi: 10.1007/s00436-026-08778-z (PMC13690864; doi:10.1007/s00436-026-08778-z)
Supplement: Supplementary file 1 [file 436_2026_8778_MOESM1_ESM.docx]

***Supplementary Figure S1***

***Figure S1: Gel electrophoresis of Nested PCR.*** *Patient blood sample positive both for P. falciparum and P. ovale****. Line 1****: patient Dna sample with P. falciparum specific primers;* ***Line 2****: negative control,* ***Line 3****: P. falciparum positive control,* ***Line 4****: patient Dna sample with P. vivax specific primers;* ***Line 5****: negative control;* ***Line 6****: P. vivax positive control;* ***Line 7****: patient Dna sample with P. malariae specific primers;* ***Line 8****: negative control****; Line 9****: P. malariae positive control;* ***Line 10*** *patient Dna sample with P. ovale specific primers;* ***Line 11****: negative control;* ***Line 12****: P. ovale positive control.*
